# Simulation atomic force microscopy for atomic reconstruction of biomolecular structures from resolution-limited experimental images

**DOI:** 10.1371/journal.pcbi.1009970

**Published:** 2022-03-16

**Authors:** Romain Amyot, Arin Marchesi, Clemens M. Franz, Ignacio Casuso, Holger Flechsig

**Affiliations:** 1 Aix Marseille University, CNRS, INSERM, LAI, Turing Centre for Living Systems, Marseille, France; 2 Nano Life Science Institute (WPI-NanoLSI), Kanazawa University, Kakuma-machi, Kanazawa, Ishikawa, Japan; Virginia Tech, UNITED STATES

## Abstract

Atomic force microscopy (AFM) can visualize the dynamics of single biomolecules under near-physiological conditions. However, the scanning tip probes only the molecular surface with limited resolution, missing details required to fully deduce functional mechanisms from imaging alone. To overcome such drawbacks, we developed a computational framework to reconstruct 3D atomistic structures from AFM surface scans, employing simulation AFM and automatized fitting to experimental images. We provide applications to AFM images ranging from single molecular machines, protein filaments, to large-scale assemblies of 2D protein lattices, and demonstrate how the obtained full atomistic information advances the molecular understanding beyond the original topographic AFM image. We show that simulation AFM further allows for quantitative molecular feature assignment within measured AFM topographies. Implementation of the developed methods into the versatile interactive interface of the BioAFMviewer software, freely available at www.bioafmviewer.com, presents the opportunity for the broad Bio-AFM community to employ the enormous amount of existing structural and modeling data to facilitate the interpretation of resolution-limited AFM images.

## Author summary

Atomic force microscopy (AFM) allows to visualize the dynamics of single biomolecules during their activity. All observations are, however, restricted to regions accessible by a fairly big probing tip during scanning. Hence, AFM images only the biomolecular surface with limited spatial resolution, missing important information required for a detailed understanding of the observed phenomena. We employ simulation atomic microscopy (sAFM), computationally emulating experimental scanning, to automatically fit available biomolecular structures into experimental AFM images. This allows to obtain 3D atomistic molecular structures from experimental AFM surface scans. For applications ranging from single molecular machines, protein filaments, to assemblies of protein lattices, we demonstrate how the full atomistic information advances the molecular understanding beyond experimental images. The developed computational methods are embedded within the user-friendly BioAFMviewer interactive software interface, providing the convenient platform for the Bio-AFM community to employ simulation AFM to better understand experimental results.

## Introduction

Nowadays nanotechnology allows to observe how single proteins work. Under atomic force microscopy (AFM), e.g., the protein surface can be scanned to image functionally important conformations [1–5]. The development of high-speed atomic force microscopy (HS-AFM) allowed to even visualize the conformational motions of proteins under near physiological conditions [6–10]. While HS-AFM has become a leading technique to study dynamical processes in single biomolecules, a complete understanding of functional mechanisms from imaging alone is not possible. This is mainly because the scanning tip can probe only the molecular surface in the scanning direction, missing the detection of all other conformational dynamics, and the spatial resolution does not typically allow to visualize structural details below the nanometer scale. Although, recently reported methods of post-imaging data processing demonstrate resolutions at the sub-nanometer scale [11], observations are still confined to the conformational dynamics of the scanned surface and the interpretation of images remains generally challenging.

On the other side, high-resolution molecular structures of proteins are known [12] and conformational dynamics can be obtained from multi-scale molecular modelling [13–15]. The enormous amount of available high-resolution protein data offers the great opportunity to better understand resolution-limited AFM scanning experiments. As an important step in that direction, we have recently developed the BioAFMviewer software platform which provides an interactive interface for simulated AFM scanning of biomolecular structures and conformational dynamics [16]. The simulated scanning method computationally emulates scanning of the molecular structure to produce pseudo AFM images. It has been employed in several studies to interpret experimental AFM images of proteins [8, 17–22], and in molecular simulations of flexible fitting biomolecular structures to experimental images [23] or to deduce information on the AFM tip shape [24].

The strengths of the stand-alone BioAFMviewer software are its user-friendly versatile interface and rich functionality. The visualization of molecular structures and simulated scanning to display corresponding AFM graphics proceeds in a synchronized way, even for very large protein structures. Scanning parameters such as tip-shape and spatial resolution, as well as the displayed range of topography heights, can be conveniently adjusted. Furthermore, molecular movies of conformational motions can be processed, which in principle allows simulated AFM experiments of functional dynamics in biomolecules.

Here we report the developed computational framework which employs simulated AFM scanning to obtain 3D atomistic structural information from resolution-limited AFM surface scans by applying automatized rigid-body fitting of biomolecular structures to AFM images. We also present a toolbox of methods for detailed analysis and comparison of surface topographies in simulated and real AFM images, which allows molecular feature assignment and further validation of experimental observations. We demonstrate the broad applicability of the developed methods for AFM images ranging from single molecular machines, protein filaments, to large-scale 2D protein lattices. As we will show, the obtained full atomistic information clearly advances the molecular understanding beyond the original topographic AFM images.

## Design and implementation

Our report shall highlight the applications of the developed computational methods to AFM images and demonstrate the achieved improvements in understanding experimental observations. For a detailed explanation of the simulated AFM scanning method and its implementation into the BioAFMviewer software, we refer to our previous report [16]. The developed strategies of automatized fitting of structures to AFM images are explained with the details provided in S1 Text.

### Fitting biomolecular structures to AFM images

The core improvement of the BioAFMviewer is a toolbox which implements optimal fitting of high-resolution biomolecular stuctures to resolution-limited experimental AFM images. The purpose of this application is to extract information on the three-dimensional molecular structure from just the surface topography available from real AFM images.

**Fig 1:**
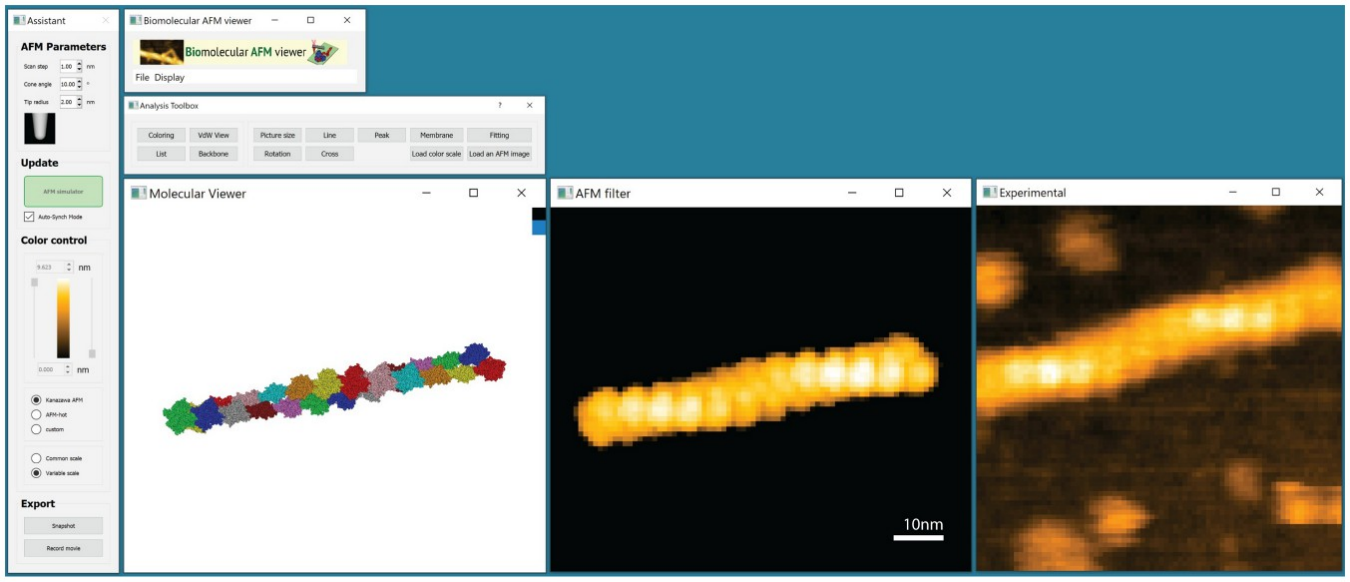
BioAFMviewer interactive interface. Central are the synchronized molecular viewer and AFM windows which provide live simulated scanning of the loaded molecular structure in arbitrary orientations. The *Assistant* window (left) allows to conveniently change scanning parameters such as spatial resolution and tip shape, and to adjust the height range and color scale of the displayed simulated AFM graphics. The *Toolbox* provides the implemented topography analysis tools and the tool for fitting to an uploaded experimental AFM image (right). Here, the actin filament was used for demonstration.

Generally, fitting of a molecular structure to an AFM image can be viewed as a complex optimization problem of identifying, from the pool of all possible conformations, a subset for which simulated and experimental AFM image match best. The interactive BioAFMviewer interface, shown in Fig 1, allows the straight-forward design and implementation of a simplified fitting procedure. The synchronized live scanning of the instantaneous orientation of a biomolecular structure displayed in the molecular viewer allows us to directly compare (in principle) *all possible* corresponding simulated AFM graphics to the target experimental AFM image uploaded by the user. The fitting strategy should therefore aim to efficiently sample the space of possible rigid-body orientations of the loaded biomolecule, score the similarity of the corresponding simulated AFM to the user-defined target AFM image, and select candidates with show the best match.

We developed a procedure which employs two search strategies to identify molecular structures which best match the target AFM image. A method of *Global Search* was based on extensive unbiased structure sampling, while the *Local Search* methods provides a more sophisticated search algorithm for refined optimization.

#### Global search strategy

The *Global Search* method relies on unbiased sampling orientations of the loaded structure in the molecular viewer. The search space was defined by rigid-body rotations around all three spatial axes within the coordinate system of the molecular viewer in discrete steps (see S1 Text). Similar to the usual workflow of the BioAFMviewer, each sampled molecular structure undergoes simulated AFM scanning to compute an AFM image that can be compared with the experimental target image. A variety of scoring functions are provided to quantitatively evaluate their similarity (see S1 Text). After completed fitting the user can conveniently access results in the *Fitting Window*. The five molecular structures with the highest scores are presented together with their corresponding simulated AFM images. The reason to provide not only the best matching structure with the highest similarity score as the single optimal fit, is that it may not necessarily represent the visually best comparison to the target AFM image. This is mainly because of the limited spatial resolution of AFM images and the approximate method of simulated scanning, which generally prevents a unique result that can be granted full trust. Particularly for proteins with symmetric domain arrangements ambiguities can be expected. We therefore provide a candidate choice from top fits.

We note that the *Global Search* method is similar to that recently reported for the rigid-body fitting to HS-AFM data [19]. In that regard, our independent work distinguishes itself by the convenient implementation into the user-friendly BioAFMviewer software interface (see Discussion). Apart from that, our approach to fitting was aimed to go beyond rather inefficient unbiased search, providing faster implementations described next.

#### Local search—Quick fit function

While the global search function samples molecular structures along a grid of globally available orientations, we also aimed to provide a more sophisticated fitting method employing a refined search strategy. The Quick Fit function performs a local search of rigid-body orientations confined to the neighborhood of a given initial molecular orientation, and identifies a single molecular structure which best fits the target AFM image. We applied a computationally efficient method which resembles a simplified Metropolis algorithm of optimization (see S1 Text). The initial molecular orientation can be, e.g., the result of a search by hand, during which the simulated AFM image of the instantaneous structure displayed in the molecular viewer is visually compared to the target experimental image. We have previously demonstrated that manual search can reasonably well predict atomic protein structures from experimental HS-AFM images [16]. In that regard the *Quick-Fit* function can serve as the speed-run solution to the complex fitting endeavour, as we will show in the Results section. On the other hand, the initial orientation may correspond to a candidate fit obtained from global search. In both situations, the Quick Fit function can be used to obtain a further refinement of structure fitting.

#### Tip-shape dependence

In our previous report we have discussed how the tip-shape geometry affects simulation AFM images [16]. Parameters characterizing the tip shape should play a role in the optimization process of finding the best match between simulation and experimental AFM image. Recent studies of structural fitting to AFM images showed that optimal parameters can be determined only within a margin of uncertainty [19, 24], which is due to the limited resolution of the imaging method. Within the BioAFMviewer interface the user can easily modify the tip-shape and observe the immediate effect on the computed AFM image, and, as we have shown previously, simulation AFM images which remarkably well reproduce experimental AFM images can thus be obtained [16]. Therefore, to avoid heavy computation, our *Global Search* procedure is performed with preset tip shape parameters. For the much faster *Quick Fit* algorithm, however, we allow to perform independent runs, each time with a different combination of probe radius and cone angle varied around the preset tip shape. In this report we will not elaborate on the effect of the tip-shape on fitting, as this was systematically explored previously [19, 24].

### Topography analysis tools

Image comparison methods provide a quantitative assessment of the overall similarity between simulated and experimental AFM images. Beyond that, it is also desirable to have tools available which allow a comparison on a more detailed level, e.g., those analyzing the height topography of the scanned biomolecular surfaces. For the analysis of biological images convenient software plugins within the ImageJ platform are routinely employed [25].

We have implemented the *Line tool*, *Cross tool*, and *Peak tool*, which all enable the user to conveniently select specific regions for which topography analysis shall be conducted within simulated and experimental AFM images.

### Membrane tool

AFM is often applied to study conformational dynamics in proteins which operate inside membranes, such as transporters or channels [26–29]. In this case the protein is embedded into a membrane-like lipid bilayer structure which is assembled on top of the scanning surface. Scanning is therefore performed for the combined membrane protein system.

We have implemented the *Membrane tool* to roughly mimic such a situation. In a chosen molecular orientation the user can add a solid double layer of adjustable width which is placed parallel to the scanning surface at a specified distance. Those parameters can be chosen to approximately take into account the relative position of the lipid bilayer for the investigated transmembrane protein.

## Results

### Application to experimental AFM images

To demonstrate the implemented algorithms of optimal fitting, we applied it to reconstruct the atomistic 3D molecular structure of the cyclic nucleotide-gated ion channel SthK from the observed AFM surface scanning images. HS-AFM imaging of this channel reconstituted into bilayer membranes revealed the formation of 2D lattices [26].

**Fig 2:**
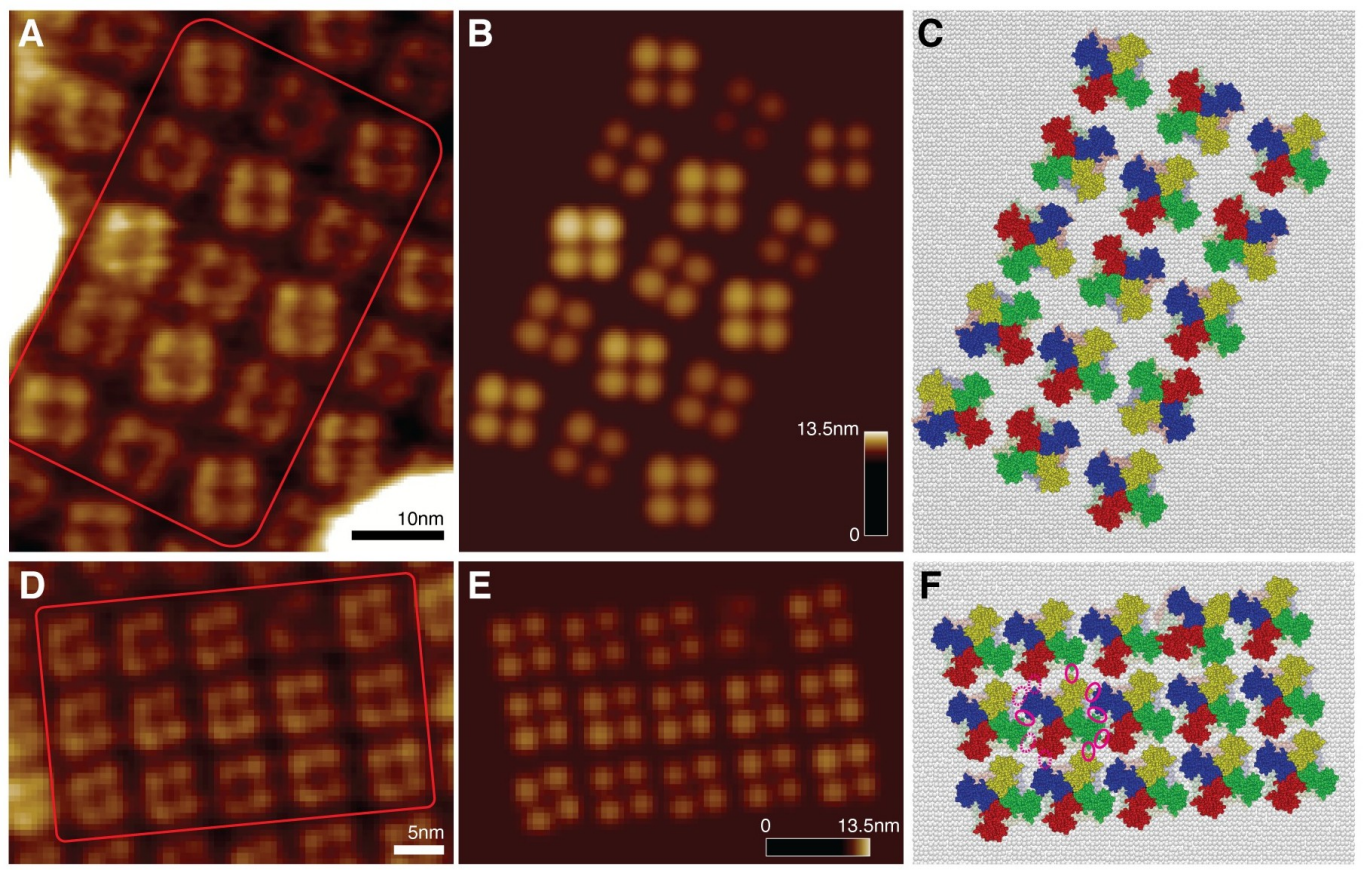
Optimal fitting to AFM images. A,D: HS-AFM images of the 2D lattice formation of SthK channels obtained from scanning the extracellular side in the activated state (A), and resting state (D), respectively. B,E: Simulated AFM images obtained from optimized fitting the molecular channel structure (PDB 6CJQ) individually into 15 selected AFM surface scans for each lattice (marked by red frames in A,D). C,F: Arrays of corresponding atomistic SthK structures for the activated state (C), and resting state lattice (F), respectively. For clarity the extracellular regions and voltage sensing domains are displayed in opaque colors, while intracellular domains are transparent (colors represent different domains in the tetramer). The lipid bilayer is schematically illustrated in gray. For a selected channel in the resting state lattice (F), the contact interfaces of its voltage sensing domains with that of neighboring channels are marked by pink ellipses using solid (tight interactions) and dashed (membrane-mediated interactions) borders.

We considered two AFM images obtained by scanning the lattice from the extracellular side under different physiological conditions. One scan corresponded to imaging the array of SthK channels in their resting state, where binding of the cyclic adenosine monophosphate (cAMP) ligand was inhibited by the presence of the competitive antagonist cGMP, whereas the other scan monitored channel conformations in the activated state after the supply of cAMP at saturating concentration. In the experiments scanning the extracellular side, a thorough interpretation of AFM images, possibly providing a structural-level understanding of the mechanism underlying channel activation was particularly challenging, because of the tetramer structure only the rather small fraction of protruding regions could be monitored, whereas most domain parts including the important voltage-sensing domains were located within the lipid bilayer and therefore inaccessible. It should be noted that neither the use of a sharper AFM tip, nor a higher spatial resolution, would resolve those issues. For both AFM surface scans (shown in Fig 2A and 2D) we performed optimal fitting to retrieve the atomistic structure of 15 individual SthK conformations from a selected array.

In practice fitting was executed one-by-one, each time considering the same atomic SthK template structure (PDB 6CJQ) individually for all target single channel topographies within the experimental array. To determine the optimal match between a simulated AFM image of the SthK structure and the particular experimental target image, a combination of *Global Search* using a coarse searching grid, and refined *Local Search* was always employed (see S1 Text). In Fig 2B and 2E the simulated images after completed optimization are shown. They compare remarkably well to their respective experimental AFM target images, quantified by very high overall comparison scores (see S1 Table). The corresponding atomistic structures are shown in Fig 2C and 2F. Regarding the tip-shape dependence, we found that fitting results were overall not sensitive to small changes in parameter values, evidenced by negligible dispersion in relative similarity scores.

The reconstruction of atomistic SthK structures, including the conformations of transmembrane domains which were not accessible to the scanning tip during experimental imaging, allows to understand important aspects underlying the mechanism of channel activation beyond what was possible from the original topographic AFM images. The lattice representing the resting state reveals a densely packed arrangement of symmetrically aligned channel conformations with a molecular orientation in which the voltage-sensing domains (VSDs) of neighboring channels can form tight surface contacts (marked in Fig 2F). Within such an environment conformational motions of channels corresponding to equilibrium fluctuations are highly confined within a *quasi-frozen* state, which is confirmed by HS-AFM movies [26]. The situation is very much different for the lattice representing the activated SthK states. There, individual channels are found to have an overall larger spatial separation within the lattice, and their relative orientations are much less coordinated. Such larger-scale rearrangement must emerge as a collective effect, and results from significant motions of the nucleotide-binding domains (NBDs) at the intra-cellular site in each channel, which are triggered by binding of cAMP ligands there. This has been evidenced by AFM observations of the intracellular surface of the channel lattice [26]. An important consequence, as we find, is that in their activated state the interface of VSDs became disrupted (compare Fig 2C and 2F), which reveals indirect evidence of long-range coupling between cytoplasmic NBDs and the peripheral membrane embedded VSDs.

Since reconstruction of both SthK lattices relied on the atomic structure of the inactive channel conformation (active state data is unfortunately lacking), our model cannot directly resolve changes of peripheral VSDs related to channel activation. However, the arrangement obtained for the SthK active state lattice leads us to hypothesize that such motions may indeed be involved in the activation process as a result from cAMP ligands binding at the remote intracellular NBDs. The existence of this allosteric communication provides a possible structural rationale for the relationship between ligand binding, transmembrane voltage modulation, and channel activation observed in functional assays of SthK [30] and other eukaryotic homologue channels [31, 32].

This application demonstrates well the purpose of the BioAFMviewer automatized fitting function. Employing available structural templates allows to obtain 3D atomistic conformations from AFM surface scans. Thus, within the limitations of this approach (see Discussion section), a molecular-level understanding of functional mechanisms beyond the information provided by the original topographic AFM images becomes available.

**Fig 3:**
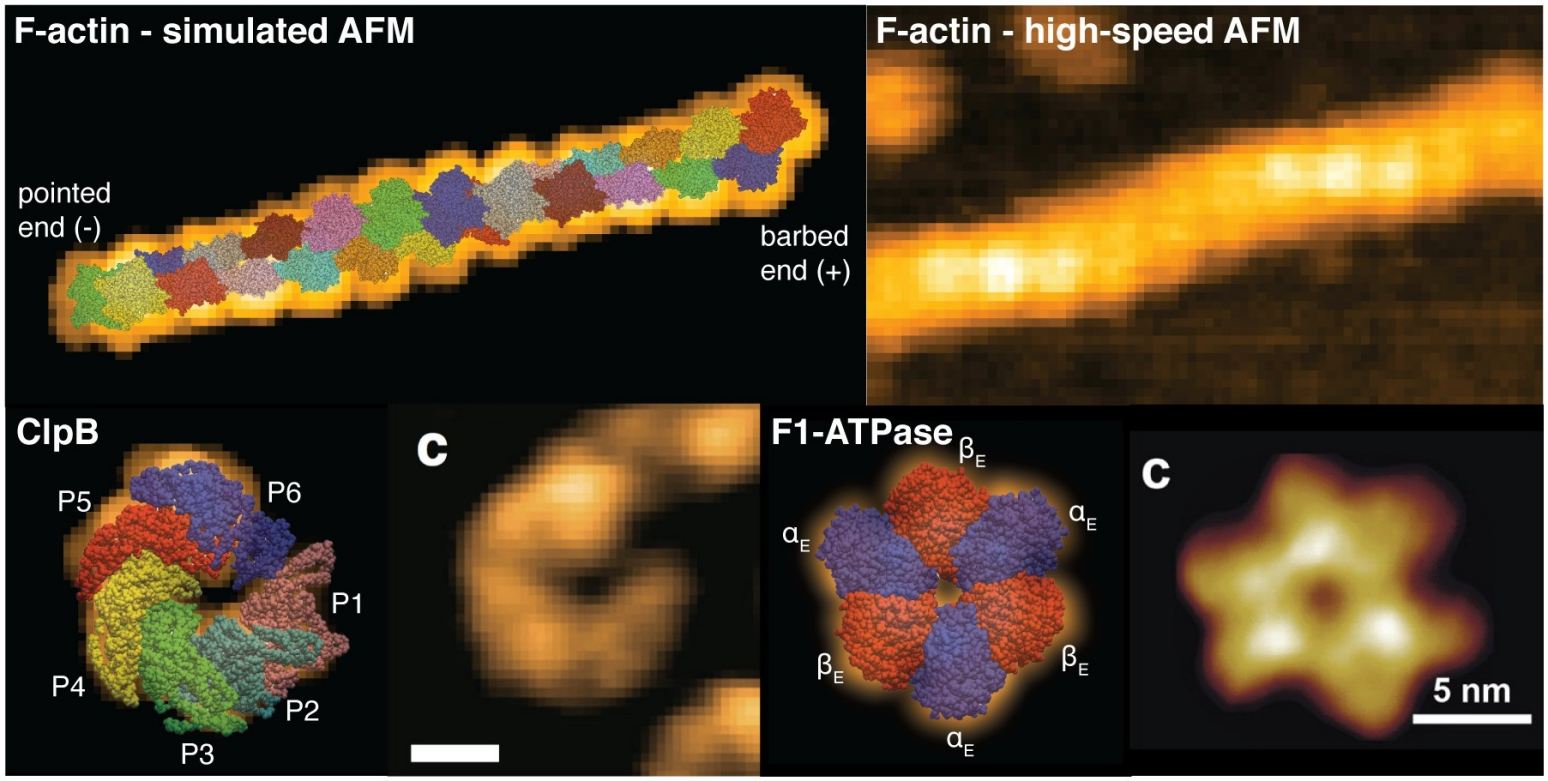
Fitting to AFM images. Top row: Superposition of the actin-filament all-atom structure and the simulated AFM image (left) obtained from fitting to the experimental HS-AFM image (right). In the molecular structure the barbed-end is located at the right side (red and blue colored domains.) Bottom row: Reconstruction of the atomistic protein structure from fitting to AFM images for the ClpB chaperone (left, PDB 5KNE), and the F1-ATPase (right, PDB 1SKY). The ClpB HS-AFM image is adopted from [17] (scale bar is 5nm) and that of F1-ATPase is from [10].

In Fig 3 further applications are provided, including fitting of the actin filament all-atom structure to an experimental HS-AFM image, and those of the ClpB chaperone and F1-ATPase motor which we have previously considered in our first report of the software [16]. Fitting to the actin filament AFM image was based on a previously constructed atomic model [33], consisting of 24 subunits (i.e. a total of ∼70.000 atoms). This application highlights the efficiency of the implemented *Quick-Fit* function. The heavy computational resources required to conduct *Global Search* would be a complete waste, given the obvious fact that the relevant search space is confined to the elongated quasi-planar orientations of the filament placed on the Mica surface under HS-AFM observations. Interestingly, the obtained result of optimized fitting for F-actin seems to allow the identification of filament polarity in the HS-AFM image from the reconstructed atomistic structure in which the barbed- and pointed-end is clearly distinguished (Fig 3, top panels). The ClpB chaperone and F1-ATPase motor are prime examples that demonstrate the explanatory strength of AFM simulations and structural fitting to disambiguate the arrangement of functional domains seen in AFM images (Fig 3, bottom panels).

Remarkably, simulation AFM also allows to confirm the nucleotide-state of domains from surface scans. This information becomes available from the PDB template used for fitting. For the ClpB chaperone the used PDB structure contained all domains in the ATP-bound state (PDB 5KNE). In the case of F1-ATPase the all nucleotide-free structure (PDB 1SKY) was used for fitting, validating the symmetric AFM topography. As we have previously shown, fitting of a partially nucleotide-complexed atomic F1-ATPase structure to an experimental HS-AFM image obtained under ligand saturation conditions allowed to identify the domain which was in the unliganded conformation [16].

### Application of topography analysis tools

**Fig 4:**
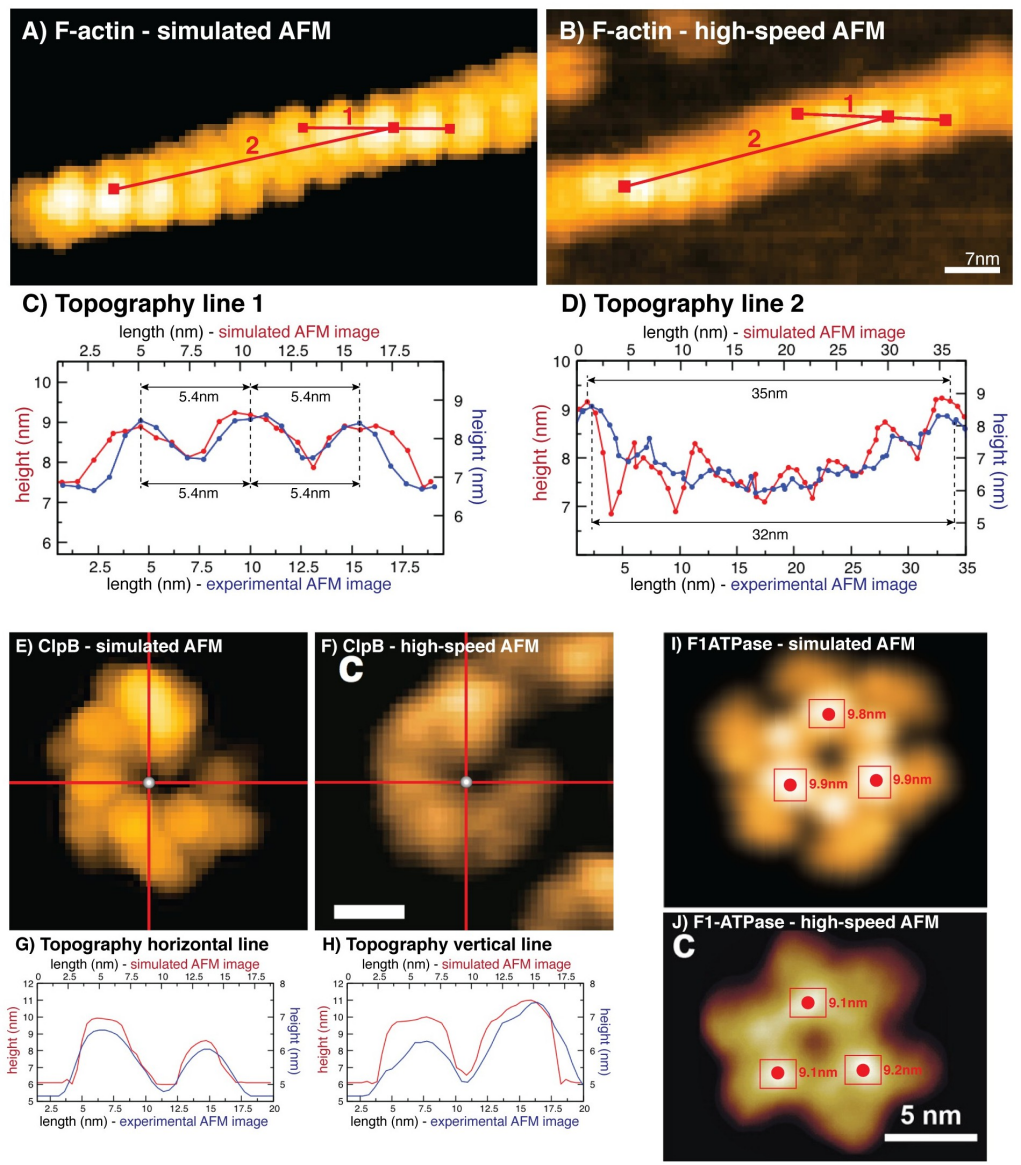
Demonstration of the Topography Tools. A-D: Line Tool function applied to the simulated and experimental AFM image of F-actin (A,B). The two chosen lines are displayed in red. Their corresponding height topographies obtained from simulated and AFM scanning of the molecular surface are compared in panels (C) and (D). E-H: Cross Tool applied to the ClpB protein. Height profiles along vertical and horizontal red lines chosen in simulated (E, using PDB 5KNE) and experimental AFM images (F, adopted from [17]; scale bar is 5nm) are compared in panels (G) and (H). I,J: Peak Tool applied to F1-ATPase images. In both simulated (I, using PDB 1SKY) and AFM graphics (J, adopted from [10]), user-selected regions are marked by red rectangles, inside which red spheres indicate positions with the largest surface height (corresponding values are given).

A demonstration of topography tools is provided in Fig 4. The *Line Tool* allows the user to draw an arbitrary line in the window canvas of the simulated AFM graphics and that of the uploaded AFM image. The height topography of the scanned molecular surface along the chosen line is then computed and displayed. As a demonstration, this tool was applied to analyze surface topographies of filamentous actin (F-actin) obtained from simulated scanning (Fig 4A) and from experimental high-speed AFM observations (Fig 4B, see S1 Text). Measuring the consecutive arrangement of filament incorporated actin monomers (line 1) shows a very good agreement of simulated and experimental images, both revealing a spatial separation between neighboring monomers units of about 5.4nm (Fig 4C). Comparing the line profiles obtained over larger distances (line 2) also shows a very good agreement of the scanned periodic arrangement along the filament structure (Fig 4D), with the half-helical pitch measured as ∼35nm and ∼32nm. This is compatible within the evident approximations of simulated scanning, and further taking account that in the experiment the actin filament was stabilized by phalloidin molecules which may alter the native structure.

To demonstrate the *Cross Tool* and *Peak Tool* functions we consider examples from our previous publication [16] and now provide further quantitative analysis of fitted simulated images to HS-AFM images. The *Cross Tool* allows the user to navigate the origin of a cross formed by horizontal and vertical lines along the canvas of simulated AFM and experimental AFM window, upon which the profiles of scanned surface heights along the corresponding cross sections are computed and displayed. Fig 4E and 4F present this tool applied to simulated and HS-AFM image of the ClpB chaperone protein [17]. The height profiles given in Fig 4G and 4H show a well agreement of relative surface heights in simulated and AFM images. Finally, the *Peak Tool* allows to specify rectangular shaped regions in the canvas of the simulated AFM and experimental AFM window, inside which the location of the highest protrusion value is detected. Application of the *Peak tool* to simulated and HS-AFM images of the nucleotide-free conformation of the rotorless F1-ATPase protein motor [10] (Fig 4I and 4J) show a very well agreement.

The performed analysis of AFM topographies for various proteins, demonstrating remarkable agreement of computational and real surface scanning, further emphasizes the important role of simulation AFM to quantitatively validate experimental data. It is worth to note that for the given examples, simulated scanning produced surface heights of systematically larger magnitude when compared to those obtained from experimental images. We will remark on this in the Discussion section where limitations of the developed computational methods are explained.

### Application of membrane tool

**Fig 5:**
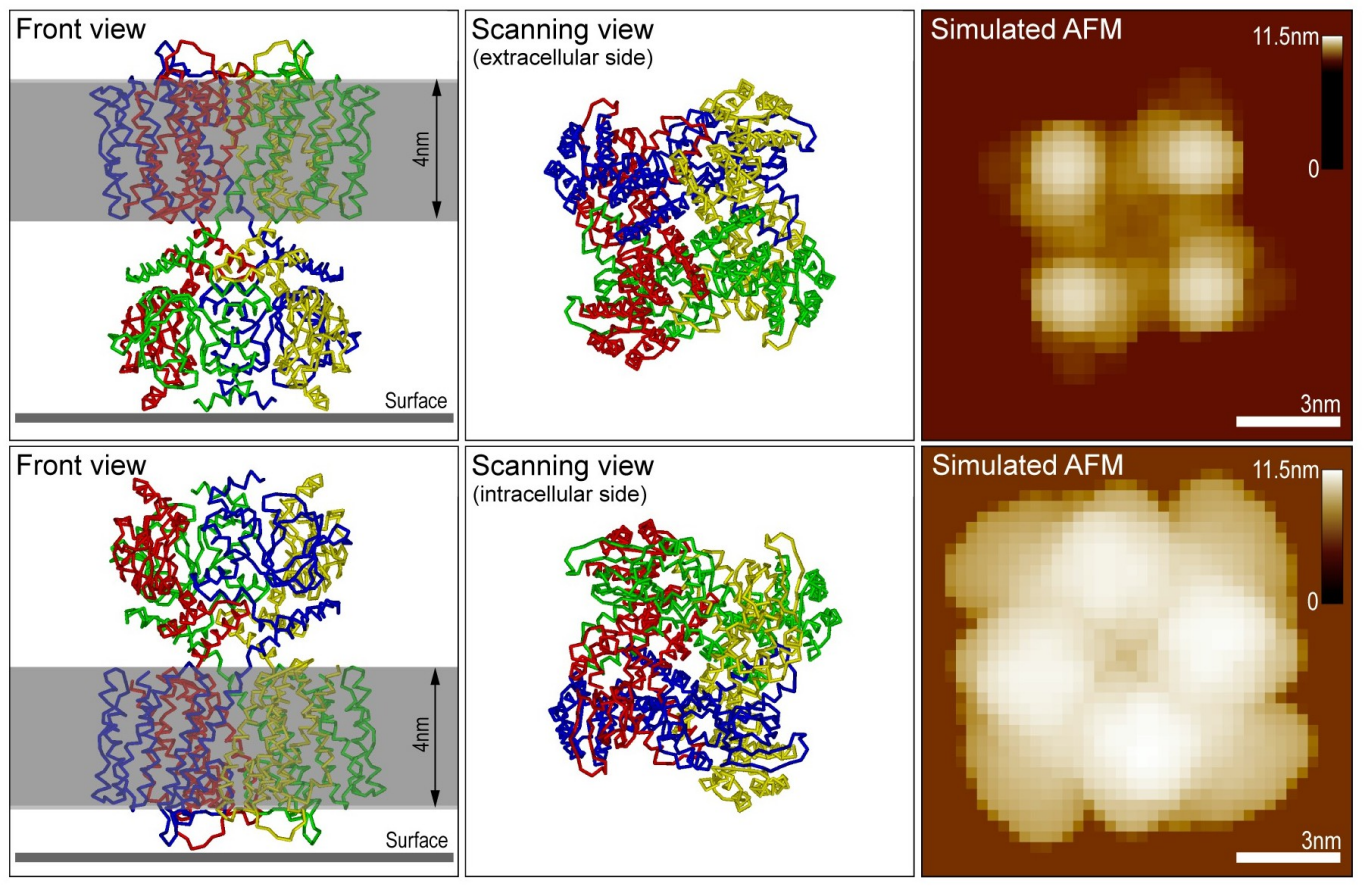
Demonstration of the Membrane Tool. Simulated scanning of the SthK ion channel (PDB 6CJQ) from the extracellular side (top row), and the intracellular side (bottom row). Channel structures in the orientations in which scanning is performed from the top are shown in the middle panel. The corresponding front view perspectives (left panel) display the placement of the solid double layer. On the right side the simulated AFM images are shown together with the respective color bars of detected height for the channel double-layer system.

In Fig 5 we provide a demonstration of the *Membrane Tool* using the PDB structure of the SthK ion channel (PDB 6CJQ). Simulated scanning was performed from the extracellular and the opposing intracellular side. The orientation of corresponding structures was conveniently chosen in the molecular viewer (Fig 5, middle panel). A proper placement of the double layer with width 4nm was obtained using the perspective shown in the *Front View* window (Fig 5, left panel). Its distance to the scanning surface was adjusted such that the layer surrounds the known transmembrane region of SthK. Simulated AFM images for this protein-membrane model system are provided in the right panel of Fig 5.

### BioAFMviewer 2 and online video manuals

Since the BioAFMviewer software is already used by many AFM groups worldwide, and valuable response helping to improve its functionality has been received, we use this opportunity to mention some of the important improvements we implemented in the BioAFMviewer 2 version. The loading of large PDB files, displaying the molecular structures, and, computing the corresponding simulated AFM images, has been highly accelerated to enable a more efficient handling of large protein structures. The integrated molecular viewer now also provides the option to view the loaded structure in the *Backbone View*, in addition to the standard Van-der-Waals (VdW) view. This representation helps in the recognition of secondary structure motifs when biomolecules are compared and fitted to AFM images. However, the user should be aware that simulated scanning is obviously always performed for the atomistic VdW representation. For the graphical representation of the simulated AFM image we included an option to upload customized color palettes according to which topography heights are translated into displayed colors.

To illustrate the workflow within the BioAFMviewer platform as easy-to-follow steps we have recorded video manuals which are available on the BioAFMviewer YouTube channel https://www.youtube.com/channel/UCKdeegdAX_ak6DlCs33x9Yg. The first manual explains the general usage, following the path from loading PDB files, to visualization of the molecular structure and the simulated AFM images, the adjustment of scanning parameters and the displayed range of surface heights including the color palette, and, eventually, the export of obtained results as image and movie files. The second manual proceeds with the application of automatized fitting molecular structures to uploaded experimental AFM images, showing a typical workflow in which a combination of *Global Search* and the *Quick Fit* function is executed. The third video manual demonstrates applications of the topography analysis tools. The illustration of the *Membrane tool* is available in a separate video manual.

## Discussion

We report simulation AFM as a computational framework to obtain 3D atomistic information from resolution-limited topographic AFM images by applying automatized rigid-body fitting of biomolecular structures, and provide the convenient implementation of developed methods into the BioAFMviewer software platform.

Applications ranging from single molecular machines, protein filaments, to large-scale assemblies of protein lattices, demonstrate how the obtained full atomistic information advances the molecular understanding beyond the original AFM topography image. We have shown that the molecular arrangement of functional domains in multimeric proteins and even their nucleotide-state can be disambiguated from AFM surface scans. In particular, applications to AFM images of 2D lattices for the cyclic nucleotide-gated SthK channel stand out to exemplify the explanatory power of atomic reconstruction. Structural characterization of the transmembrane domains, which are not accessible to the scanning tip during imaging, allows to identify possible interactions sites between neighboring channels within the membrane and elucidate the pattern distinguishing the resting state from the activated state.

Of major importance to us was that the computational procedure of simulated AFM scanning together with the developed mathematical methods of fitting and topography analysis are embedded within a well organized user-friendly graphical interface. In that regard, the integrated molecular viewer, synchronized computation and visualization of AFM graphics, convenient adjustment of scanning and model parameters, and export of obtained results are central. It distinguishes AFM simulations from previous implementations.

The rigid-body fitting to AFM images was recently presented in another work [19], where also the tip-shape geometry was parameterised to obtain best agreement to an experimental image (available as a script file afmize without a graphical interface). While our *Global Search* method is similar, the fitting approach presented here was aimed to go beyond inefficient exhaustive unbiased search. The interactive BioAFMviewer interface allowed us to implement the *Quick Fit* function for highly efficient fitting to AFM images, which can also consider the tip-shape dependence on fitting. As a further distinction, the BioAFMviewer interface allows applications beyond static PDB files. Simulation AFM can be conducted for entire molecular movies, e.g., obtained from modeling simulations [16]. This opens the opportunity to employ data of functional conformational dynamics for fitting to experimental AFM images [22].

Its user-friendly environment and rich functionality establishes the BioAFMviewer software package as a convenient platform for the broad Bio-AFM community to employ the enormous amount of existing structural and modelling data to facilitate the interpretation of resolution-limited AFM images.

Application of simulation AFM can reach much beyond just the interpretation of what is observed in an AFM image. We have previously shown that new structural models with atomistic resolution can be retrieved for higher oligomeric arrangements of the Annexin V protein seen under HS-AFM [20]. Simulation AFM was recently applied in a demonstration study of a membrane transporter protein, showing that based on topographic imaging of only one membrane side, the functionally coupled conformational state at the remote opposite site can be predicted [22].

The explanatory power of simulation AFM to predict correlated conformational dynamics in proteins from topopgraphic AFM imaging [22] will play a major role in the future to complement AFM experiments.

### Availability, limitations, and future directions

Simulated AFM scanning employed in the BioAFMviewer can only approximate the complex experimental scanning process, and its limitations have been discussed in detail in our previous report [16]. An important aspect is that simulated scanning, based on non-elastic collisions of the tip with the molecular surface, apparently produces surface heights of systematically larger magnitude compared to invasive experimental scanning. Apart from that, as we demonstrate, the agreement of relative heights of the scanned proteins is remarkable.

While more sophisticated formulations of simulated scanning are possible, e.g., resolving tip-sample interactions at a higher level of detail, this would correspond to a significant increase in computational resources and prevent the smooth implementation of synchronized AFM simulations. More importantly, applications to AFM images of biomolecular structures clearly evidence the superb performance of approximate modelling.

The presented fitting method also has limitations. It treats the loaded PDB structure as a rigid object which cannot undergo internal conformational changes. The fitting quality to an experimental AFM image is therefore confined to simulation AFM of possible rigid-body molecular orientations. Our applications show indeed that fitting of PDB templates works very well for AFM images of proteins displayed in equilibrium conformations. As mentioned above, BioAFMviewer applications using molecular modelling data can overcome this drawback.

In this report, we have not considered cases of fitting fragmented structural data into AFM images. This situation becomes relevant for the interpretation of HS-AFM images of highly flexible proteins with intrinsically disordered regions [34, 35], or of proteins complexed with nucleic acids [36]. In this case available PDB data can still be used for partial reconstruction of the imaged biomolecular structure. The upcoming BioAFMviewer distribution shall implement a convenient procedure which allows fitting of structural templates to preassigned image sections within the target AFM image.

A possible future objective is to provide a script-driven batch processing of the simulation AFM calculations, which can automatize the comparison of movie frames obtained from molecular simulations to those from HS-AFM videos.

## Supporting information

S1 Text Details of the fitting procedure, applications to HS-AFM images, and computational benchmark are provided. (PDF)

S1 Table Similarity scores between simulated and target AFM image for fitting of the SthK channel atomistic structure to the 15 selected AFM surface scans representing the activated state (A), and resting state (B), respectively. (PDF)
