# Supplementary material for: Simulation atomic force microscopy for atomic reconstruction of biomolecular structures from resolution-limited experimental images
Source: PLoS Comput Biol. 2022 Mar 16;18(3):e1009970. doi: 10.1371/journal.pcbi.1009970 (PMC8959186; doi:10.1371/journal.pcbi.1009970)
Supplement: S1 Text — (PDF) [file pcbi.1009970.s001.pdf]

Simulation atomic force microscopy for atomic reconstruction of biomolecular structures from resolution-limited experimental images – Supporting Information

Romain Amyot[1], Arin Marchesi[2,3], Clemens M Franz[2], Ignacio Casuso[1], and Holger Flechsig[2,*]

[1]Aix Marseille University, CNRS, INSERM, LAI, Turing Centre for Living Systems, Marseille, France
[2]Nano Life Science Institute (WPI-NanoLSI), Kanazawa University, Kakuma-machi, Kanazawa, Ishikawa, Japan
[3]Current address: Dipartimento di Medicina Sperimentale e Clinica, Università Politecnica delle Marche, Ancona, Italy
[*]flechsig@staff.kanazawa-u.ac.jp

S1 Text

Materials and methods

Fitting biomolecular structures to AFM images

Quantitative comparison of simulated and experimental AFM images

During the optimization process the agreement of simulated to target AFM image was scored taking into account a variety of quantitative similarity measures.

The image correlation coefficient (ICC) is based on the pair-wise comparison of information encoded in the pixels. It was computed as $C = (\sum_{i,j}(p_{ij}^{sim} - \bar{p}^{sim}) \cdot (p_{ij}^{exp} - \bar{p}^{exp}))/\sqrt{\sum_{i,j}(p_{ij}^{sim} - \bar{p}^{sim})^2 \cdot \sum_{i,j}(p_{ij}^{exp} - \bar{p}^{exp})^2}$,

On the other side one can compute the image cosine similarity which neglects the pixel information relative to the average and instead considers just absolute values, i.e., $C = \sum_{i,j} p_{ij}^{sim} \cdot p_{ij}^{exp} / \sqrt{\sum_{i,j}(p_{ij}^{sim})^2 \cdot \sum_{i,j}(p_{ij}^{exp})^2}$.

We also employed the root mean square deviation of pair-wise pixel information (image RMSD), computed as $\mathrm{RMSD} = \sqrt{\frac{1}{N}\sum_{i,j}(p_{ij}^{sim} - p_{ij}^{exp})^2}$.

We note that the pixel information p_{ij} refers to the pixel color intensity.

Details of Global Search

For unbiased sampling of the structure loaded in the molecular viewer, the search space of molecular orientations was obtained by executing rigid-body rotations around all three spatial axes within the coordinate system of our molecular viewer in discrete steps. It was given by the set of discrete orientations $\mathscr{O}_{ijk} = (\alpha_i, \beta_j, \gamma_k)$ characterized by three angles $\alpha_i, \beta_i, \gamma_i = i \cdot \Omega$, where Ω is the angular grid spacing. This spacing parameter can be specified by the user, such that 360° is a multiple of Ω. If, for example, $\Omega = 10^\circ$ is chosen, the number of sampled structures in the fitting procedure would be $36^3 = 46656$.

The user can choose to run the global search optimization with either of provided correlation scores or combinations of them computed at the same time. We recommend activating all of them. After completed fitting, the user can access the results conveniently from a switch panel, upon which the corresponding fitted molecular structure together with the simulated AFM graphics is displayed in their separate windows, ready to be visually compared to the target experimental AFM image.

Details of Local Search

Local search optimization samples structures in an iterative process of small amplitude rigid-body rotations followed by selection, to eventually identify a single molecular structure best fitting the target AFM image. Starting from the initial structure with orientation $\mathscr{O}^{\mathrm{ini}} = (\alpha^{\mathrm{ini}}, \beta^{\mathrm{ini}}, \gamma^{\mathrm{ini}})$, a next candidate structure with changed orientation $\mathscr{O}^{\mathrm{cand}} = (\alpha^{\mathrm{ini}} + \delta\alpha, \beta^{\mathrm{ini}} + \delta\beta, \gamma^{\mathrm{ini}} + \delta\gamma)$ is obtained by allowing a rigid-body rotation with angle changes $\delta\alpha, \delta\beta$, and $\delta\gamma$. Those angle changes were randomly drawn

from a gaussian distribution with center zero and a width value that can be specified by the user. The new candidate structure was then evaluated by scoring the agreement of simulated to target AFM image. Only if the candidate structure improved the agreement it was accepted to replace the initial orientation, i.e. $\mathscr{O}^{\text{cand}} = \mathscr{O}^{\text{ini}}$, while otherwise it was rejected. The next iteration cycle starts either from the improved fit, or samples other structures around the previous one. The fitting process is terminated when the improvement reached a sufficient level of saturation.

It can be noted, that the implementation of the local search procedure is a simplified variant of the Metropolis algorithm of optimization (namely the zero temperature limit, or steepest descent). By selecting only candidate structures which improve agreement scores, the applied method provides an efficient approximation to identify the best fitting structure in the neighborhood of the initial molecular orientation.

Application to Hs-AFM images

For AFM scans of SthK channels in the activated and resting state, fitting was based on simulated scanning of the molecular PDB structure 6CJQ. From the AFM image of the lattice 15 single channel topographies within the selected array were manually clipped first. After that, fitting of the molecular structure was performed one-by-one for each target AFM clipping individually. It consisted of applying *Global Search* with a grid spacing of $\Omega = 40^\circ$ followed by *Quick Fit* with a variance of 1nm. The parameters used in simulation AFM were $R = 2$nm for the tip sphere radius and 10° (5°) for the cone-half angle. The chosen spatial resolution corresponded to the experimental value of 0.33nm/pixel and 0.57nm/pixel for the activated and resting state lattice, respectively. In S1 Table we provide a list of similarity scores of simulated to target AFM image obtained for the individual fittings.

For fitting to the AFM image of the actin filament we used the atomic model Actin_model.pdb provided in reference[2]. The scanning parameters were $R = 1.5$nm, 10° and 0.77nm/pixel. Fitting was obtained by applying the *Quick Fit* function with a variance of 1nm, starting from a manually chosen molecular conformation.

For fitting to the AFM image of the ClpB chaperone we used the PDB structure 5KNE with scanning parameters $R = 1$nm, 10° and 0.4nm/pixel. In the case of F1-ATPase we used the 1SKY structure and parameters $R = 1$nm, 10° and 0.3nm/pixel.

Benchmark details

The BioAFMviewer performance was tested on a standard laptop machine, namely a Lenovo-ThinkPad with Intel(R) Core(TM) i7-8850H CPU @ 2.60GHz 2.59GHz (6 cores and 64GB RAM). As for the speed of the optimization procedure we provide approximate values obtained with the specific chosen parameters for scanning and fitting. For SthK the *Global Search* fitting of an individual channel ($\sim$12.770 atoms) typically took about 3min and the subsequent *Quick Fit* was always completed in less than half a minute. *Quick-Fit* of the actin filament model ($\sim$70.500 atoms) was completed within 2 minutes. Fitting of the ClpB structure ($\sim$14.500 atoms) was comparable to the SthK case. For the F1-ATPase structure ($\sim$21.900 atoms) fitting was completed within a few minutes.

Experimental high-speed AFM images

The SthK channel 2D lattice was obtained from imaging SthK proteins reconstituted in bilayer membranes that were adsorbed on a mica surface. Imaging was performed at a spatial resolution of 0.33nm/pixel and 0.57nm/pixel for the activated and resting state, respectively. Image tilt correction and horizontal line-by-line leveling as described in[3] was additionally conducted.

The image of actin was obtained from scanning a phalloidin-stabilized filament on a Mica surface that was treated with (3-aminopropyl)triethoxysilane (APTES). The total scanning area of 60×60 nm^2 was monitored over a grid of 78×78 pixels^2, which defined the spatial resolution we applied for simulated scanning.

References

1. Orzechowski M, Tama F. Flexible fitting of high-resolution x-ray structures into cryelectron microscopy maps using biased molecular dynamics simulations. Biophys J. 2008; 95: 5692-5705. https://doi.org/10.1529/biophysj.108.139451

2. Pirani A, Vinogradova MV, Curmi PMG, King WA, Fletterick RJ, Craig R, et al. An atomic model of the thin filament in the relaxed and Ca2+-activated states. J Mol Biol. 2006; 357: 707-717. https://doi.org/10.1016/j.jmb.2005.12.050

3. Zuttion F, Redondo-Morata L, Marchesi A, Casuso I. High-resolution and high-speed atomic force microscopy imaging. Yuri L Lyubchenko. Nanoscale Imaging. Methods and protocols, 1814, Springer, pp.181-200, 2018, Methods in Molecular Biology, 978-1-4939-8590-6. https://doi.org/10.1007/978-1-4939-8591-3_11
