# Supplementary material for: Simulation atomic force microscopy for atomic reconstruction of biomolecular structures from resolution-limited experimental images
Source: PLoS Comput Biol. 2022 Mar 16;18(3):e1009970. doi: 10.1371/journal.pcbi.1009970 (PMC8959186; doi:10.1371/journal.pcbi.1009970)
Supplement: S1 Table — (PDF) [file pcbi.1009970.s002.pdf]

# Simulation atomic force microscopy for atomic reconstruction of biomolecular structures from resolution-limited experimental images – Supporting Information

**Romain Amyot[1], Arin Marchesi[2,3], Clemens M Franz[2], Ignacio Casuso[1], and Holger Flechsig[2,*]**

[1]Aix Marseille University, CNRS, INSERM, LAI, Turing Centre for Living Systems, Marseille, France
[2]Nano Life Science Institute (WPI-NanoLSI), Kanazawa University, Kakuma-machi, Kanazawa, Ishikawa, Japan
[3]Current address: Dipartimento di Medicina Sperimentale e Clinica, Università Politecnica delle Marche, Ancona, Italy
[*]flechsig@staff.kanazawa-u.ac.jp

# S1 Table

**A**

| SthK AFM scan | DCC | ICC | RMSD |
|---|---|---|---|
| 1 | 0.94 | 0.89 | 17.91 |
| 2 | 0.85 | 0.78 | 13.99 |
| 3 | 0.90 | 0.80 | 19.32 |
| 4 | 0.89 | 0.86 | 18.32 |
| 5 | 0.97 | 0.94 | 15.77 |
| 6 | 0.87 | 0.80 | 18.85 |
| 7 | 0.94 | 0.84 | 33.51 |
| 8 | 0.89 | 0.76 | 23.94 |
| 9 | 0.96 | 0.93 | 18.82 |
| 10 | 0.87 | 0.58 | 26.93 |
| 11 | 0.95 | 0.92 | 21.64 |
| 12 | 0.94 | 0.86 | 15.68 |
| 13 | 0.94 | 0.82 | 24.20 |
| 14 | 0.92 | 0.91 | 15.65 |
| 15 | 0.96 | 0.90 | 15.97 |

**B**

| SthK AFM scan | DCC | ICC | RMSD |
|---|---|---|---|
| 1 | 0.92 | 0.66 | 16.80 |
| 2 | 0.93 | 0.66 | 14.41 |
| 3 | 0.93 | 0.71 | 14.99 |
| 4 | 0.97 | 0.85 | 6.06 |
| 5 | 0.93 | 0.74 | 16.26 |
| 6 | 0.92 | 0.64 | 19.34 |
| 7 | 0.93 | 0.65 | 18.23 |
| 8 | 0.93 | 0.62 | 18.55 |
| 9 | 0.93 | 0.73 | 16.81 |
| 10 | 0.96 | 0.75 | 14.52 |
| 11 | 0.92 | 0.58 | 20.49 |
| 12 | 0.93 | 0.64 | 17.00 |
| 13 | 0.93 | 0.68 | 15.11 |
| 14 | 0.93 | 0.70 | 16.87 |
| 15 | 0.94 | 0.70 | 17.45 |

S1 Table: Similarity scores between simulated and target AFM image for fitting of the SthK channel atomistic structure to the 15 selected AFM surface scans representing the activated state (A), and resting state (B), respectively.
